# Long-term air pollution exposure and living close to busy roads are associated with COPD in women

**DOI:** 10.1186/1465-9921-6-152

**Published:** 2005-12-22

**Authors:** Tamara Schikowski, Dorothea Sugiri, Ulrich Ranft, Ulrike Gehring, Joachim Heinrich, H-Erich Wichmann, Ursula Krämer

**Affiliations:** 1 Institut für Umweltmedizinische Forschung (IUF) at the Heinrich-Heine-University of Düsseldorf, Auf'm Hennekamp50, 40225 Düsseldorf, Germany; 2 GSF – National Research Center for Environment and Health, Institute of Epidemiology, Ingolstädter Landstrasse 1, 85764 Neuherberg, Germany; 3 Ludwig-Maximilians-University of Munich, Institute of Medical Data Management, Biometrics and Epidemiology, Chair of Epidemiology, Geschwister-Scholl Platz 1, 80539 Munich, Germany; 4 Utrecht University, Institute for Risk Assessment Sciences, P.O. Box 80. 176, NL-3508 TD Utrecht, The Netherlands

## Abstract

**Background:**

Lung function and exacerbations of chronic obstructive pulmonary disease (COPD) have been associated with short-term exposure to air pollution. However, the effect of long-term exposure to particulate matter from industry and traffic on COPD as defined by lung function has not been evaluated so far. Our study was designed to investigate the influence of long-term exposure to air pollution on respiratory symptoms and pulmonary function in 55-year-old women. We especially focused on COPD as defined by GOLD criteria and additionally compared the effects of air pollution on respiratory symptoms by questionnaire data and by lung function measurements.

**Methods:**

In consecutive cross sectional studies conducted between 1985–1994, we investigated 4757 women living in the Rhine-Ruhr Basin of Germany. NO_2 _and PM_10 _exposure was assessed by measurements done in an 8 km grid, and traffic exposure by distance from the residential address to the nearest major road using Geographic Information System data. Lung function was determined and COPD was defined by using the GOLD criteria. Chronic respiratory symptoms and possible confounders were defined by questionnaire data. Linear and logistic regressions, including random effects were used to account for confounding and clustering on city level.

**Results:**

The prevalence of COPD (GOLD stages 1–4) was 4.5%. COPD and pulmonary function were strongest affected by PM_10 _and traffic related exposure. A 7 μg/m_3 _increase in five year means of PM_10 _(interquartile range) was associated with a 5.1% (95% CI 2.5%–7.7%) decrease in FEV_1_, a 3.7% (95% CI 1.8%–5.5%) decrease in FVC and an odds ratio (OR) of 1.33 (95% CI 1.03–1.72) for COPD. Women living less than 100 m from a busy road also had a significantly decreased lung function and COPD was 1.79 times more likely (95% CI 1.06–3.02) than for those living farther away. Chronic symptoms as based on questionnaire information showed effects in the same direction, but less pronounced.

**Conclusion:**

Chronic exposure to PM_10_, NO_2 _and living near a major road might increase the risk of developing COPD and can have a detrimental effect on lung function.

## Background

Acute exacerbations of chronic obstructive pulmonary disease (COPD), chronic bronchitis or emphysema have been associated with short-term exposure to air pollution [1-3]. Studies done in the 1970s found that high levels of particles were related to a high prevalence of chronic bronchitis [4,5]. However, recent studies designed to measure the effects of long-term exposure to air pollution on pulmonary function and respiratory health in adults are rare [6-10]. The studies conducted so far did not use a definition of COPD based on lung function but focused on respiratory symptoms [11].

Several studies have suggested that lung function decline and respiratory diseases are associated with proximity to roads with heavy traffic, traffic density or exposure to traffic-related air pollution [12-15]. The majority of these studies investigated the influence of air pollution on children's lung function and respiratory health. Only one study has investigated the impact of chronic traffic pollution on pulmonary function exclusively in women [16], however the focus was on FEV_1 _decline and asthma rather than on COPD.

Our study was done between 1985 and 1994 when sulfur dioxide and particle pollution from industrial sources already had decreased whereas traffic-related pollution was increasing. Women are probably more susceptible for COPD and respiratory symptoms caused by environmental factors than men, therefore the study focused on women only [17,18]. We defined COPD by lung function according to the newly developed GOLD criteria [19]. The study was designed to investigate the association between COPD as defined by lung function (FEV_1_/FVC <0.7) and chronic exposure to air pollution from industrial sources and traffic.

We compared this association with the effect of chronic exposure of air pollution on different respiratory symptoms assessed by questionnaire. Effects from air pollution were also compared to single lung function parameters FEV_1 _and FVC.

## Methods

### Study design and population

The SALIA study (Study on the influence of air pollution on lung function, inflammation and aging) was part of the Environmental Health surveys as an element of the Clean Air Plan introduced by the Government of North-Rhine Westphalia in Germany [20]. Consecutive cross-sectional studies were performed between 1985 and 1994. The study areas (Dortmund (1985, 1990), Duisburg (1990), Essen (1990), Gelsenkirchen (1986, 1990) and Herne (1986)) were chosen to represent a range of polluted areas with high traffic load and steel and coal industries. Two non-industrial small towns, Dülmen (1985) and Borken (1985, 1986, 1987, 1990, 1993, 1994), were chosen as reference areas. Data from similar studies done in 1987, 1993 and 1994 in Cologne, Düsseldorf, Hürth, Dormagen and Wuppertal were not included in this analysis because of a low response, different type of exposure (chemical industry) and non availability of address-coordinates for GIS- based exposure estimation.

All women aged 54 to 55 residing in the selected areas were asked to participate in the study, which took place in March and April in the years specified. 4874 responded, every second responder was invited to have a lung function testing (N = 2593). We restricted the analysis to those 4757 women whose addresses were available and where the addresses could be merged with geographic coordinates. Men were not recruited for the study, to avoid bias due to occupational exposure from working in the mining and steel industry.

### Questionnaire: diagnoses, symptoms and risk factors

Together with an invitation to participate in the study, a self-administered questionnaire was sent to the women. The investigating physicians checked the returned questionnaires. We asked whether a physician had ever diagnosed chronic bronchitis and for respiratory symptoms. Respiratory symptoms were asked as "chronic cough with: (a) phlegm production, (b) for > 3 month a year, (c) for more than 2 years". We evaluated "chronic cough" and "chronic cough with phlegm production". The diagnosis of chronic cough with phlegm production was positive, when each of the answers categories (a), (b) or (c) was positive. This symptoms complex classically defines chronic bronchitis.

We further asked about risk factors such as single room heating with fossil fuels, occupational exposure (dust and extreme temperatures) and education as indicator for socioeconomic status. We classified socioeconomic status into three categories using the highest school level achieved by either the women or her husband as low (< 10 years), medium (= 10 years) or high (> 10 years). Women were grouped according to their smoking habits as never smoker, passive-smoker (home and/or work place), past smoker or current smoker (<15 pack years; 15–30 pack years and >= 30 pack years).

### Lung function testing and COPD

Forced expiratory volume in 1 second (FEV_1_) and forced vital capacity (FVC) were measured. Four maneuvers were performed, and the values, where the maximal FEV_1 _was reached, were used. All measuring instruments were calibrated prior to each testing by using a 3-liter-syringe. All personal were specially trained, the same type of measuring device was used (Vica Test 4 spirometer (Mijnhardt, Rotterdam, Holland)) and all maneuvers were performed in accordance to a standardized protocol [21]. We also used the ratio FEV_1_/FVC, which is considered a sensitive measure of COPD on its own [22]. A FEV_1_/FVC ratio <0.7 is the main criterion for COPD according to the newly developed criteria by GOLD [19]. We used this criterion to define the disease. However, we did not use a post-bronchodilator measurement in our epidemiological study, therefore we excluded 168 women with asthma from further analysis of the association between lung function and air pollution, to avoid confounding. Asthma was considered present, when ever diagnosed by a physician or if asthma medication were used.

### Air pollution

We used two ways to assess air pollution exposure, first, we used data from monitoring stations maintained by the State Environment Agency. They cover the area in an 8 km grid and are designed to mainly reflect broad scale spatial variations in air quality. Second, we used distance of residential address to the nearest major road, which reflects small-scale spatial variations in traffic related exposure.

All 7 monitoring stations used for this study were located within a distance of not more than 8 km to the women's home address. Given that there was no monitoring station available for Dülmen, the air pollution data from Borken was used, because of its proximity and comparability. Due to the incompleteness of air pollution data from Borken, where continuous measurements started in 1990, the data preceding this year were imputed by using measurements (1981–2000) from 15 monitoring stations in the Ruhr area assuming similar trends. Between 1985 and 1987 discontinuous measurements were performed in Borken and Dülmen (four days per month). These discontinuous measurements agreed well with the imputed values. Mean measured TSP between 1984–1987 was 70 μg/m^3 ^and the imputed value for 1985 was 66 μg/m^3^.

The concentrations of nitrogen dioxide (NO_2_) was measured half-hourly by means of chemiluminescence. Total suspended particles (TSP) were gathered with a low volume sampler (air flow: 1 m^3^/h) and measured using beta-ray absorption. For the assessment of individual medium term air pollution exposure we used annual mean concentrations in the year of the investigation and for long-term air pollution exposure we used five-year means of measurements done before the investigation. To estimate the exposure of particulate matter of less than 10 μm dynamic diameters (PM_10_), we multiplied TSP measurements with a conversion factor of 0.71. This conversion factor was calculated from 7 monitoring sites in the Ruhr area, where parallel measurements of TSP and PM_10 _were performed between 1998 and 2004.

We further assessed the exposure to motor vehicle exhaust by the distance (< 100 m and >= 100 m) from each residential address to the nearest major road (> 10 000 cars per day) by using geographic information system (GIS) software Arc GIS 9.0 (ESRI Redlands, CA). Average daily traffic counts for the year 1997 and mean traffic load per square kilometer for the year 1987 (without Borken and Dülmen) were obtained from the North Rhine Westphalia State Environment Agency (LUA NRW).

### Statistical method

The association of symptoms and diagnoses with ambient air pollution exposure was analyzed by logistic regression. Odds ratios (OR) with 95% confidence intervals (CI) are presented for an interquartile range increase in PM_10 _[7 μg/m^3^] and NO_2 _[16 μg/m^3^] exposure and for living nearer than 100 m respectively >= 100 m from a road with heavy traffic. FEV_1_, FVC and the ratio FEV_1_/FVC were approximately normally distributed and multiple linear regressions were used for analysis. The regression coefficients b were transformed to relative mean differences (MD) MD = 1+b/mean (lung function). We included a random area effect in the logistic as well as the linear regression analysis to account for possible clustering within areas.

Age, socioeconomic status, smoking, exposure to environmental tobacco smoke (ETS), occupational exposure to temperature (heat/cold) and dust and heating with fossil fuels were included as covariates in all models. FEV_1 _and FVC were adjusted for body mass index (BMI) and height additionally.

All statistical analysis was done with SAS for windows release 9.1 (SAS Institute, Cary, NC).

## Results

### Description of the study population

The characteristics of the 4757 women are shown in table 1. The overall response rate was 70% (range 62%–80%), which remained stable over the years of study and showed no systematic differences between urban and rural areas over time. According to the study design, the age range was very narrow and the mean age of the women was identical 54.5 years in each year and area. The majority of women reported to be never smokers: 40.1% without exposure to environmental tobacco smoke (ETS) and 33.5 % with ETS exposure at home or at work. Occupational exposure to dust or extreme temperatures at work was reported by 11.6 % respectively 9.9%. According to our definition, 47.8% of the women or their partners had an education of at least 10 years of schooling, a medium socio-economic status (SES).

**Table 1:** Characteristics of study participants

**Participants (N = 4757)**	**n/N**	**%**	
Time of residency ≥ 5 years under			
current address	4255/4749	89.6	
Smoking status			
Never -smoker without ETS	1762/4396	40.1	
Never-smoking with ETS	1472/4396	33.5	
Ex-smoker	384/4396	8.7	
Current smoker			
<15 pack years	269/4396	6.1	
15–30 pack years	282/4396	6.4	
>30 pack years	227/4396	5.7	
Single room heating with fossil fuels	1039/4653	21.8	
Occupational exposure to dust/fumes	552/4757	11.6	
Occupational exposure to extreme temperatures	469/4757	9.9	
Social status			
Low	1401/4702	29.8	
Medium	2248/4702	47.8	
High	1051/4702	22.4	
	**N**	**mean**	**SD**

Age [years]	4755	54.5	0.6
Body Mass Index [kg/m^2^]	3844	27.7	4.7
Height [cm]	3846	162.1	5.8

The prevalence of doctor-diagnosed chronic bronchitis was 9.5% and frequent cough was reported by 22.5% of the women and chronic cough with phlegm production was reported by 4.6% (table 2). The diagnosis of bronchitis was less frequently reported from women who participated in the spirometric measurements compared to women who did not participate. Differences in symptom prevalence between these groups could not be detected. The prevalence of COPD defined by the criterion FEV_1_/FVC <0.7 was 4.5%.

**Table 2:** Prevalence of airway diseases, symptoms and lung function in 55 year old women

	**all**		**With spirometry (N = 2593)**		
	**n/N**	**%**	**N/N**	**%**	

Chronic bronchitis by physician diagnosis	442/4649	9.5	211/2537	8.4	
Chronic cough with phlegm production	225/4701	4.8	116/2563	4.6	
Frequent cough	1065/4731	22.5	561/2581	21.8	

COPD FEV_1_/FVC<0.7			116/2581	4.5	

			**n**	**Mean**	**SD**

FEV_1 _[L]			2590	2.55	0.46
FVC [L]			2584	3.09	0.51
FEV_1_/FVC			2581	0.83	0.07

### Air pollution exposure

18.5% of all women lived in a distance of less than 100 m from a road with more than 10 000 cars a day (major road). Medium distance was 494 m (table 3). Correlation (Pearson's r) of mean traffic load per km^2 ^between 1987 and 1997 was r = 0.7.

**Table 3:** Distribution of air pollution exposure

N = 4757	**Min**	**P 25**	**Median**	**Mean**	**P 75**	**Max**
**Annual Mean**						
NO_2 _[μg/m^3^]	20	29	41	39	45	60
PM_10 _[μg/m^3^]	35	40	43	44	47	53
						
Distance to Road [m] with >10,000 cars/Day	6	424	494	519	556	6374

**Five year Mean**						
NO_2 _[μg/m^3^]	22	25	46	39	49	55
PM_10 _[μg/m^3^]	39	43	47	48	53	56

The distributions of annual mean and five-year mean of air pollution exposure are also presented in table 3. The range of PM_10 _was smaller than that of NO_2 _and, the ranges of the five-year means were smaller than those of the annual means. The five year means were somewhat higher than the annual means, but highly correlated (Pearson r > 0.9). Living near a major road was associated with mean values of NO_2 _but not with the other pollutants. There were considerable correlations between the single air pollutants. Pearson's r for the five year means of PM_10 _and NO_2 _was r = 0.7.

### Association between small scale ambient air pollution exposure and respiratory morbidity and lung function

Table 4 shows the results of the logistic and linear regression analysis for the association of living near a major road and respiratory diagnoses, symptoms and lung function. Women living within a radius of 100 m to a major road reported more frequent cough (adj. OR= 1.24; 95% CI 1.03–1.49). The odds ratio for the association of cough with phlegm production was greater than one, but not significant (OR 1. 07, 95% CI 0.83–1.37). The odds ratio for the association of COPD and living close to busy roads was higher and significant (OR 1.79, 95%CI 1.06–3.02).

**Table 4:** Distance to major roads and exposure to air pollutants (annual means and five year means) as predictors for respiratory symptoms and pulmonary function

	**Annual means**			**Five year means**	
	**<100 m from major road wi 10,000 cars/day compared to > 100 m**	**NO_2 _[16 μg/m^3^]**	**PM_10 _[7 μg/m^3^]**	**NO_2 _[16 μg/m^3^]**	**PM_10 _[7 μg/m^3^]**

	**OR (95% CI)**	**OR (95% CI)**	**OR (95% CI)**	**OR (95% CI)**	**OR (95% CI)**

Chronic bronchitis by physician diagnosis (n_1 _= 4205, n_5 _= 3761)	1.15 (0.89–1.50)	1.25(*) (1.00–1.58)	1.00 (0.85–1.18)	1.37** (1.16–1.62)	1.13 (0.95–1.34)
Chronic cough with phlegm production (n_1 _= 4237, n_5 _= 3792)	1.07 (0.83–1.37)	1.11 (0.85–1.45)	1.03 (0.87–1.23)	1.22 (0.90–1.64)	1.11 (0.93–1.31)
Frequent cough (n_1 _= 4262, n_5 _= 3813)	1.24* (1.03–1.49)	1.13* (1.01–1.27)	1.01 (0.93–1.10)	1.15(*) (0.99–1.33)	1.05 (0.94–1.17)

COPD FEV_1_/FVC<0.7 (n_1 _= 2314, n_5 _= 2096)	1.79* (1.06–3.02)	1.39** (1.20–1. 63)	1.37(*) (0.98–1.92)	1.43** (1.23–1.66)	1.33* (1.03–1.72)

	**MD (95% CI)**	**MD (95% CI)**	**MD (95% CI)**	**MD (95% CI)**	**MD (95% CI)**

FEV_1 _(n_1 _= 2315, n_5 _= 2095)	0.987* (0.962–0.997)	0.961** (0.939–0.984)	0.953* (0.916–0.989)	0.951** (0.925–0.977)	0.949** (0.923–0.975)
FVC (n_1 _= 2310, n_5 _= 2092)	0.982* (0.966–0.998)	0.974** (0.954–0.993)	0.966* (0.940–0.992)	0.966** (0.945–0.987)	0.963** (0.945–0.982)
FEV_1_/FVC (n_1 _= 2314, n_5 _= 2096)	0.999 (0.990–1.007)	0.989** (0.985–0.993)	0.989(*) (0.978–1.000)	0.988** (0.982–0.993)	0.989* (0.980–0.997)

Effect estimates adjusted for are age, smoking, SES, occupational exposure and form of heating FEV_1 _and FVC were additionally adjusted for BMI a height

n_1 _sample size of all women, n_5 _sample size of women living at least five years at their residence, women living less than five years at their residence were excluded in the analyses of five year means of air pollutants

(*) p < 0.1; * p < 0.05; ** p < 0.01

Women living within a radius of 100 m to a major road had a significantly decreased FEV_1 _and FVC. Although COPD as defined by FEV_1_/FVC < 0.7 was associated with distance to a major road, the ratio FEV_1_/FVC by itself was not associated with distance to major road.

Since smoking is the strongest risk factor for the development of respiratory symptoms and COPD, we repeated the analysis separately for smokers and non-smokers. The relationship between distance to major road and the development of respiratory symptoms including COPD did not change substantially (data not shown).

Additionally we repeated the analysis with distance to major road as a continuous variable (log_2 _distance), however, the pattern of the effects remained the same as with distance in two levels.

#### Association between broad scale ambient air pollution exposure and respiratory morbidity and lung function

The associations with medium-term exposure (annual means) were evaluated for all women, the associations with long-term exposure for women living at least 5 years at their place of residence (N = 4255). The odds ratios for the association between annual or five year means of air pollution and respiratory morbidity were all above one. Chronic bronchitis and frequent cough were significantly associated with NO_2 _and COPD was significantly associated with all pollutants investigated (table 4, fig. 4). All odds ratios for five-year exposure were stronger than those for one-year exposure (table 4). This was not due to the selection of women who lived more than 5 years at their residence, because the odds ratios for annual means did not change when restricting the analysis to women with a residency > 5 years.

**Figure 1:**
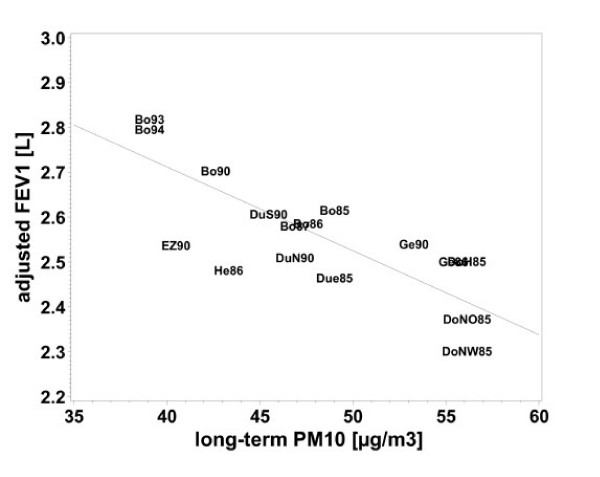
Association between FEV_1 _and long-term PM_10 _exposure (five-year mean), adjusted for age, height, BMI, SES, heating with fossil fuels, occupational exposure (Dust/ temperature) and smoking for women who lived at least five years at their place of residence. Means of each place and year of study: Bo = Borken, DoH = Dortmund Hörde, DoNO = Dortmund North-East, Due = Dülmen, DuS = Duisburg South, DuN = Duisburg North, EZ = Essen Centre, Ge = Gelsenkirchen, He = Herne

**Figure 2:**
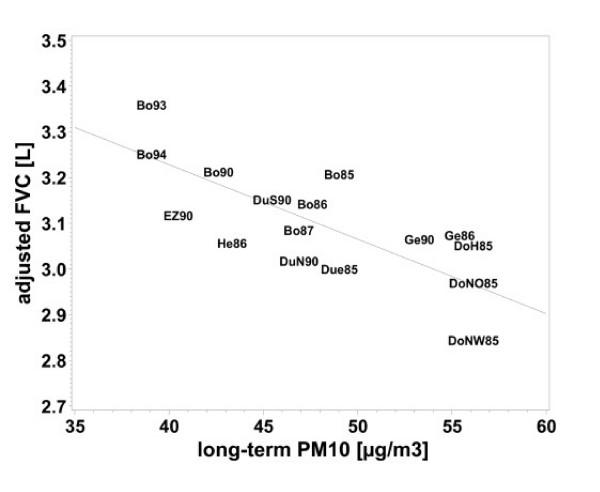
Association between FVC and long-term PM_10 _exposure (five-year mean), adjusted for age, height, BMI, SES, heating with fossil fuels, occupational exposure (Dust/ temperature) and smoking for women who lived at least five years at their place of residence. Means of each place and year of study: Bo = Borken, DoH = Dortmund Hörde, DoNO = Dortmund North-East, Due = Dülmen, DuS = Duisburg South, DuN = Duisburg North, EZ = Essen Centre, Ge = Gelsenkirchen, He = Herne

**Figure 3:**
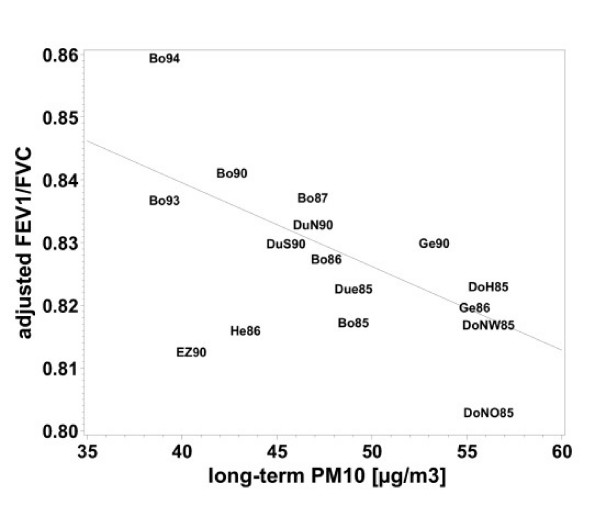
Association between FEV_1_/FVC and long-term PM_10 _exposure (five-year mean), adjusted for age, SES, heating with fossil fuels, occupational exposure (Dust/ temperature) and smoking for women who lived at least five years at their place of residence. Means of each place and year of study: Bo = Borken, DoH = Dortmund Hörde, DoNO = Dortmund North-East, Due = Dülmen, DuS = Duisburg South, DuN = Duisburg North, EZ = Essen Centre, Ge = Gelsenkirchen, He = Herne

**Figure 4:**
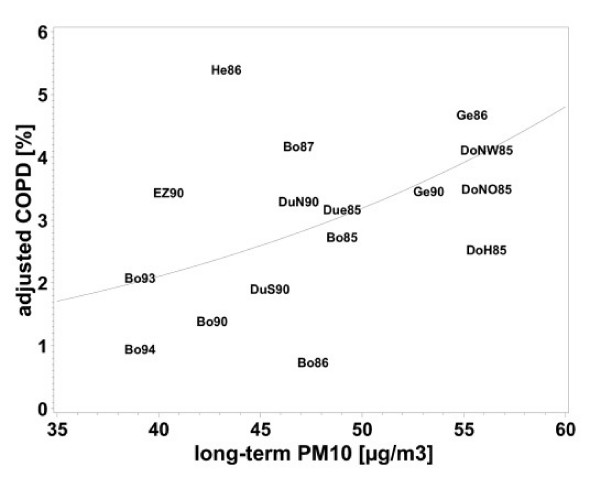
Association between COPD and long-term PM_10 _exposure (five-year mean), adjusted for age, SES, heating with fossil fuels, occupational exposure (Dust/ temperature) and smoking for women who lived at least five years at their place of residence. Means of each place and year of study: Bo = Borken, DoH = Dortmund Hörde, DoNO = Dortmund North-East, Due = Dülmen, DuS = Duisburg South, DuN = Duisburg North, EZ = Essen Centre, Ge = Gelsenkirchen, He = Herne

Linear regression revealed significant negative associations of all air pollutants with FEV_1_, FVC and FEV_1_/FVC (table 4). Again the effects were stronger for the five-year means than for the annual means (table 4). Figures 1, 2, 3, 4 demonstrate the steady decrease of lung function with increasing PM_10_.

We repeated the analysis separately for smokers and non-smokers to assess whether the effect of long term exposure to air pollutants was modified by smoking. However, no signs of interaction could be detected (data not shown).

Furthermore we conducted a sensitivity analysis in which the interaction of time with socioeconomic status and environmental tobacco smoke was tested. No change of effect could be observed for the association of these covariates with the outcomes (data not shown). We also tested whether the association between respiratory outcomes in exposure levels varies. Therefore we divided the exposures into three categories. There was a tendency of stronger association in the higher exposure category, however, the differences were not significant.

## Discussion

In this cross sectional study on 55-year-old women we found, that long-term exposure with air pollution from industrial sources and traffic had an adverse effect on pulmonary function, COPD and respiratory health. The effects on respiratory health symptoms were strongest for NO_2 _and traffic exposures. The effects of air pollutants were substantial: a 7 μg/m^3 ^change in five year means of PM_10 _was associated with a 5.1% decrease in FEV_1_, a 3.7% decrease in FVC and a 33% increase in prevalence of COPD. We found stronger effects associated with five-year means than with annual means, which is probably due to their greater stability. The associations between respiratory outcomes were slightly higher in higher exposure categories, but the differences between the categories showed no significance.

It is plausible that there is a change in the effects of covariates during the observation period, however, this seems not to be the case, because the interaction used to test this assumption was not significant.

COPD and chronic cough with phlegm production (symptoms of chronic bronchitis) were not very common in this group of 55 year old women (prevalence 4.5% and 4.6%), but for this age group similar prevalence have been found in other studies [23,24].

The pollutant results can be compared with the findings from the Swiss SAPALDIA study, which investigated the association between air pollution and respiratory health in 20–60 year old adults[25,26]. A 10 μg/m^3 ^increase of annual mean PM_10 _was associated with a 3.4% decrease in FVC and a 1.6% decrease in FEV_1 _[6]. These results point in the same direction as our results, although we found stronger effects. Contrary to us, the results presented for the SAPALDIA study were restricted to the group of healthy non-smokers. However, in the Swiss study as well as in our study the effect of PM_10 _on lung function was equally pronounced in smokers and in non-smokers. We explored whether the higher mean concentrations of PM_10 _in our study could account for this. Yet in our study the effect estimates did not depend on the absolute level of air pollution. An analysis done for the years 1985–1987 when air pollution was higher yielded similar results as an analysis with the 1988–1994 values (data not shown).

The stronger effects in our study can probably be explained by differences in the study population. We investigated 55 year old women (age range 51.9–56.3). It has already been demonstrated that the effect of smoking on lung function and COPD is stronger in women than in men [16], and this may also apply for PM_10 _effects.

A qualitative comparison can be made with a Japanese study. Sekine et al. reported a reduction in lung function parameters in females living near trunk roads [16]. In our study, we found that women living less than 100 m from a major road had an elevated risk of developing chronic cough and COPD. Living <100 m away was significantly associated with a decline in lung functions parameters and the development of COPD compared to women who lived >100 away.

Chronic bronchitis was also more prevalent in adults from Germany, living at extremely or considerably busy roads [27]. Nevertheless the associations with chronic bronchitis found in the present study were smaller, which is probably due to the differences in the study design. Several limitations of this study must be considered. One limitation is the incompleteness of air pollution measurements. Values for Borken before 1990 were imputed assuming similar trends as in the other areas. This assumption seems plausible because similar trends in Borken and the other areas have been shown for the years after 1990 and the discontinuous measurements of TSP in 1984–1987 agreed well with the imputed values. The idea of monitoring air pollution by the State Environment Agency is to survey broad scale exposure hence the 8 km grid of the monitoring stations. Therefore traffic related exposure was additionally estimated as distance of residential address to major road. However, the location of major roads may have changed between 1985–1994 and 1997, but the correlation of mean traffic load per km^2 ^in 1987, a measure available for the big cities, with the same measure in 1997 is 0.7, demonstrating proportionality of traffic over time. A further limitation is the cross sectional design of our study, where migration may cause a problem. However, this does not apply to our study, since only 10% of women moved in the last 5 years before the investigation. It is also possible, although unlikely, that some women already died from COPD or other particle related diseases before the age of 55. This could have led to an underestimation of the true effect.

The advantage of this study is the wide number of cross-sections with a large range of exposure that was included. This makes the results less susceptible to random variation in one area and year. Another advantage is the objective exposure assessment on individual level by using GIS data. Main advantage is the use of an objectively measured outcome variable namely COPD as defined by lung function and not relying on questionnaire based symptoms only.

## Conclusion

The GOLD criteria, namely the ratio FEV_1_/FVC >0.7 was useful to determine an association between air pollution and respiratory health outcomes. Hereby, it showed that COPD, as defined by lung function, provides a more evident picture of the association than the definition by symptoms only. To our knowledge this is the first study assessing long-term effects of air pollution on the development of COPD by combining broad and small-scale spatial exposure. The results of this study suggest that long-term exposure to air pollution from PM_10_, NO_2 _and living near a major road might increase the risk of developing COPD and can have a detrimental effect on lung function. However, what precisely drives this association has to be clarified in other types of study.

## Competing interests

The author(s) declare that they have no competing interests.

## Authors' contributions

T Schikowski performed the epidemiological analysis, drafted and wrote the paper. D Sugiri was co-investigator of the repeated cross-sectional studies, performed Geographical Information System analysis and was responsible for the data management and statistical analysis. U Krämer was main investigator of the repeated cross-sectional studies, commented and advised on exposure assessment statistical analysis and commented on the manuscript. U Ranft was co-investigator of the repeated cross-sectional studies and commented on the draft. HE Wichmann commented on the draft. J Heinrich commented on the draft. U Gehring provided assistance with the data management, imputed air pollution data for Borken and commented on the draft. All authors gave final approval to the version to be published.
